# The role of human Shu complex in ATP-dependent regulation of RAD51 filaments during homologous recombination–associated DNA damage response

**DOI:** 10.1016/j.jbc.2025.110212

**Published:** 2025-05-08

**Authors:** Sam S.H. Chu, Guangxin Xing, Hong Ling

**Affiliations:** Department of Biochemistry, University of Western Ontario, London, Ontario, Canada

**Keywords:** Shu complex, ATPase, DNA damage tolerance response, homologous recombination, RAD51 filament

## Abstract

Error-free DNA lesion bypass is an important pathway in DNA damage tolerance. The Shu complex facilitates this process by promoting homologous recombination (HR) to bypass DNA damage. Biochemical analysis of the human Shu complex homolog, hSWS1-SWSAP1, offers valuable insights into the HR-associated DNA damage response. Here, we biochemically characterized the human Shu complex and examined its interactions with RAD51 filaments, which are essential in HR. Using fluorescence polarization assays, we first revealed that hSWS1-SWSAP1 preferentially binds DNA with an exposed 5′ end in the presence of adenine nucleotides. We then investigated and validated the DNA-stimulated ATPase activity of hSWS1-SWSAP1 through site-specific mutagenesis, revealing that DNA with an exposed 5′ end is the most efficient in enhancing this activity. Furthermore, we showed that hSWS1-SWSAP1 initially interacts with RAD51 filaments at the 5′ end and modulates the properties of the nucleoprotein filaments using fluorescence-based assays. Our findings revealed that hSWS1-SWSAP1 induces conformational changes in RAD51 filaments in an ATP hydrolysis-dependent manner, while its stabilization of the filaments depends on ATP binding. This work provides mechanistic insights into the regulation of RAD51 filaments in HR-associated DNA damage tolerance.

Endogenous and environmental factors can induce 10^4^–10^5^ DNA lesions per mammalian cell per day, threatening genomic integrity and cell survival (1). Lesions that block DNA replication and persist into S and G_2_ cell cycle phases require a DNA damage response with homologous recombination (HR) to bypass and eventually repair the lesions (2). This error-free pathway uses sister chromatids for template-directed repair to ensure accurate replication of genetic information, minimizing disease outcomes such as cancers (2, 3). RAD51, a highly conserved protein from yeast to human, plays an essential role in facilitating HR by forming nucleoprotein filaments on single-stranded DNA (ssDNA) (3, 4). Subsequent HR events, such as homology search, strand invasion, and strand exchange with the homologous sister chromatids, are highly regulated by a group of proteins known as RAD51 mediators (2, 3, 5, 6).

The human Shu complex, hSWS1-SWSAP1, belongs to the group of RAD51 mediator proteins (2) and is a heterodimer, consisting of SWS1 and the SWS1-associated protein 1 called SWSAP1 (7). Like RAD51, the Shu complex is also highly conserved among eukaryotes, from yeast to humans (7, 8, 9, 10, 11). The human SWSAP1 subunit is a remote paralog of Rad51, containing Walker A and B motifs (7), which are conserved domains in RAD51-like ATPases for ATP binding and ATP hydrolysis, respectively (12, 13, 14, 15). The hSWS1 subunit of the hSWS1-SWSAP1 heterodimer has a conserved Zinc finger SWIM domain, which is vital to the DNA damage tolerance response in eukaryotes (11).

As shown biochemically, hSWS1-SWSAP1 has DNA-stimulated ATPase activity (7), which has also been identified in the yeast Shu complex (16). However, the specific role of hSWS1-SWSAP1 in HR-associated DNA damage tolerance remains unclear. Furthermore, the hSWS1–SWSAP1 complex was recently shown to bind RAD51-ssDNA filaments without displacing RAD51, forming interspersed filaments (17). However, it is unclear if hSWS1-SWSAP1 can influence the properties of RAD51 filaments as do the yeast and *Caenorhabditis elegans* Shu homologs, which alter the conformation/accessibility of the filaments and enhance their stability (9, 16, 18). Further biochemical characterization of the human Shu complex will help to elucidate the nature of the hSWS1-SWSAP1–RAD51 interaction and the role of hSWS1-SWSAP1 as a RAD51 mediator in DNA lesion bypass.

In this study, we report biochemical characterization of the human Shu complex, hSWS1-SWSAP1, to provide molecular insights into its role in HR. We demonstrated that hSWS1-SWSAP1 preferentially binds DNA substrates with exposed 5′ ends in the presence of adenine nucleotides. We characterized the ATP-binding and ATP-hydrolysis activities of hSWS1-SWSAP1 and revealed enhancement of enzymatic activity by DNA with exposed 5′ ends. Lastly, we showed that hSWS1-SWSAP1 changes the properties of RAD51-ssDNA filaments, requiring ATP binding and ATP hydrolysis for filament stabilization and conformational changes, respectively.

## **Results**

### DNA-binding preferences of hSWS1-SWSAP1

**Figure 1:**
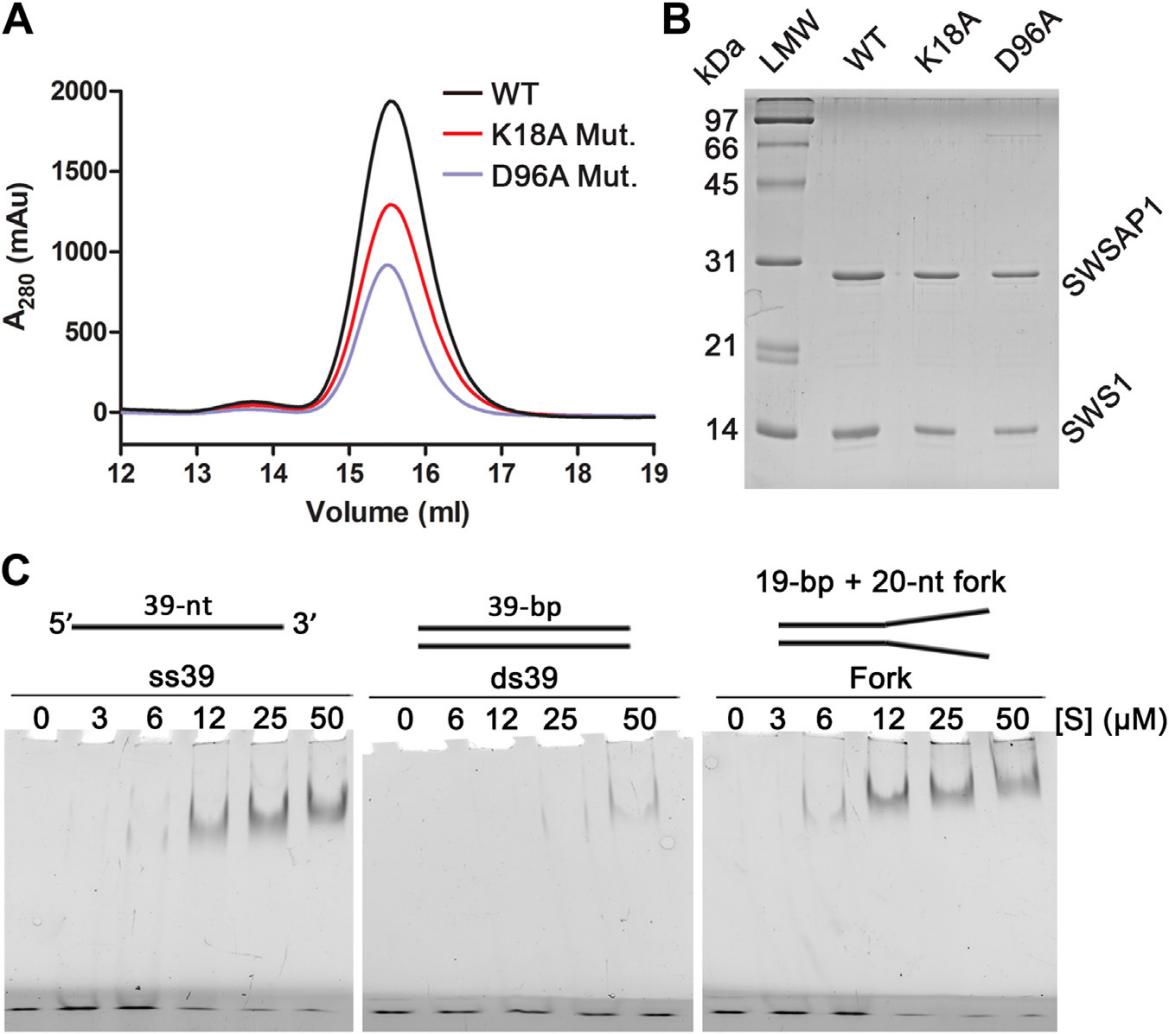
**Purified human Shu complex and its DNA binding.***A* and *B*, gel filtration (*A*) and SDS-PAGE (*B*) analysis of the purified hSWS1-SWSAP1 (wildtype, WT) and mutants (K18A and D96A in SWSAP1). *C*, electrophoretic mobility shift assay for WT hSWS1-SWSAP1–DNA interactions. Increasing concentrations of hSWS1-SWSAP1 ([S]) were mixed with 0.05 μM DNA substrates (ss39, ds39, Fork). Protein–DNA mixtures were resolved by 2-layer PAGE gels: 5% native polyacrylamide at the *top* and 15% at the *bottom* (*dark layer*). Assays were performed in triplicate with comparable results (Fig. S3, *A*–*C*). LMW, low molecular weight protein ladder.

The DNA-binding properties of the hSWS1–SWSAP1 complex are important for its role in HR-associated DNA damage tolerance. The hSWS1-SWSAP1 dimer demonstrated a general binding preference for ssDNA over blunt-ended double-stranded DNA (dsDNA) in electrophoretic mobility shift assays (EMSAs) (7). To systematically study the DNA-binding of the hSWS1–SWSAP1 complex, we carried out DNA-binding assays with a series of DNA substrates (ssDNA and dsDNA of different sizes and end types) labeled with 6-carboxyfluorescein (6-FAM) (Table 1). The hSWS1–SWSAP1 complex and its mutant variants were purified to near homogeneity with correct oligomeric sizes (Fig. 1, *A* and *B*). We first tested the DNA-binding function of hSWS1-SWSAP1 with the substrates using EMSAs in two-composition native gels: a 5% polyacrylamide gel electrophoresis (PAGE) layer at the top to allow the large DNA–protein complex to enter the gel and a 15% PAGE layer at the bottom to prevent the free DNA from running out of the gel. The results showed that hSWS1-SWSAP1 physically interacts with the substrates in a concentration-dependent manner (Fig. 1*C*). Both ssDNA and fork-shaped dsDNA shifted at lower protein concentrations than the blunt-ended ds39 substrate. This suggests a binding preference of hSWS1-SWSAP1 for ssDNA over blunt-ended dsDNA.

**Table 1:** DNA-binding affinities of hSWS1-SWSAP1

DNA	Size (nt)^a^	Sequence^b^	*K*_d_ (μM)^c^
ss21	21/-	5′- ∗ACTTACAGCACAGCGGTTTTT -3′	10.1 ± 0.9
ss26	26/-	5′- ∗ACTGCCCTTACAGCACAGCGGTTTTT -3′	9.9 ± 0.6
ss31	31/-	5′- ∗ACTGCCCTTACAGCACAGCGGTTTTTTTTTT -3′	5.7 ± 0.3
ss39	39/-	5′- TTTTTTTTTTTTTTTTTTTTCTTGACAAGCTTGCGCACT∗ -3′	11.2 ± 0.4
ds21	21/21	5′- ∗ACTTACAGCACAGCGGTTTTT -3′ 3′- TGAATGTCGTGTCGCCAAAAA -5′	43.7 ± 0.4
ds26	26/26	5′- ∗ACTGCCCTTACAGCACAGCGGTTTTT -3′ 3′- TGACGGGAATGTCGTGTCGCCAAAAA -5′	18.8 ± 0.7
ds39	39/39	5′- TTTTTTTTTTTTTTTTTTTTCTTGACAAGCTTGCGCACT∗ -3′ 3′- AAAAAAAAAAAAAAAAAAAAGAACTGTTCGAACGCGTGA -5′	19.2 ± 0.9
Fork	39/39 (20)	5′- TTTTTTTTTTTTTTTTTTTTCTTGACAAGCTTGCGCACT∗ -3′ 3′- TTTTTTTTTTTTTTTTCACGGAACTGTTCGAACGCGTGA -5′	7.7 ± 0.5
3′-overhang	39/19	5′- CTTGACAAGCTTGCGCACT∗ -3′ 3′- TTTTTTTTTTTTTTTTCACGGAACTGTTCGAACGCGTGA -5′	14.9 ± 0.7
5′-overhang	39/19	5′- TTTTTTTTTTTTTTTTTTTTCTTGACAAGCTTGCGCACT∗ -3′ 3′- GAACTGTTCGAACGCGTGA -5′	3.6 ± 0.2

a “nt” stands for nucleotide and indicates DNA-strand size; number in parentheses represent the lengths of single-stranded components of the “Fork” DNA substrate.

b Symbol “∗” denotes the position at which a fluorescein molecule (6-FAM) is attached.

c Data were obtained through nonlinear regression of the binding data as described in Experimental procedures and represent the mean ± SD (standard deviation) of three independent experiments.

Next, we determined the binding affinities of hSWS1-SWSAP1 with different DNA substrates using fluorescence polarization assays (FPAs) (Table 1 and Fig. S1, *A* and *B*). The *K*_d_ values for the substrates ranged from 3.6 to 43.7 μM, with the 5′-overhang substrate having the highest affinity and the blunt-ended dsDNA substrates having the lowest. However, the differences in *K*_d_ values were within ∼12-fold (Table 1).

**Figure 2:**
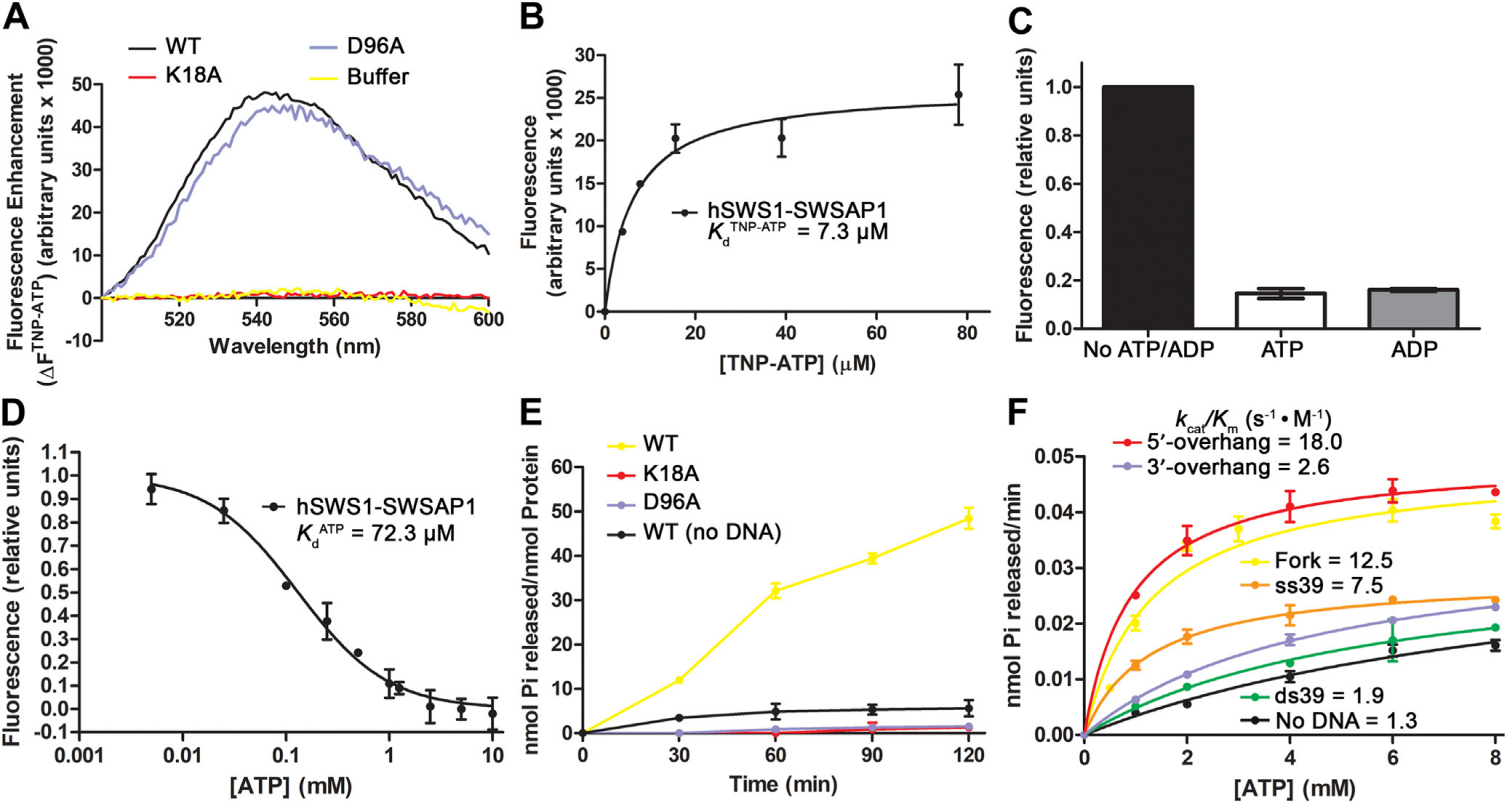
**ATP binding and ATPase activity of hSWS1-SWSAP1.***A*, fluorescence spectra of TNP-ATP (5 μM) with WT hSWS1-SWSAP1 and Walker motif mutants (K18A, D96A) (4 μM). *B*, TNP-ATP saturation assay. Increasing concentrations of TNP-ATP were added to hSWS1-SWSAP1 (2 μM). Dissociation constants (*K*_d_^TNP-ATP^) were determined by nonlinear curve fitting to a one-site binding model. *C*, competitions of TNP-ATP (5 μM)/hSWS1-SWSAP1 (4 μM) complexes by ATP or ADP (2.5 mM each). Fluorescence units were normalized to the initial readings without excess ATP and ADP. *D*, titration of TNP-ATP (2.5 μM) fluorescence with ATP in the presence of hSWS1-SWSAP1 (2 μM). Data shown represent fluorescence units after subtraction of TNP-ATP background fluorescence, followed by normalization to the reading without ATP (relative units). Data were fitted using nonlinear regression to a one-site competitive binding model. Dissociation constant (*K*_d_^ATP^) was derived from competitive binding data (see Experimental procedures). *E*, ATPase activity of WT hSWS1-SWSAP1 and Walker motif mutants (1 μM each) with DNA substrate containing a 5′-overhang (10 μM), over 120-min time course. *F*, ATPase activity (60-min reactions) of hSWS1-SWSAP1 (1 μM) in the presence of ssDNA and dsDNA substrates with different end types (1 μM each), with increasing ATP concentrations. Data were fitted using the Michaelis–Menten model to determine kinetic parameters (details found in Table 3). Data in *A–F* represent the mean of three independent replicates, with error bars in *B–F* indicating the standard deviation from the triplicate experiments. dsDNA, double-stranded DNA; ssDNA, single-stranded DNA; TNP-ATP, 2′(3′)-O-(2,4,6-trinitrophenyl) adenosine 5′-triphosphate.

We further analyzed the DNA binding of hSWS1-SWSAP1 in the presence of nucleotides (ATP and ADP) (Table 2 and Fig. S1, *C*–*E*). Surprisingly, the presence of nucleotides abolished the binding of hSWS1-SWSAP1 to blunt-ended and 3′-overhang substrates (Table 2). These dsDNA substrates lack an exposed 5′ end, which suggests that the 5′ end of DNA is likely important for the DNA binding of hSWS1-SWSAP1. In contrast, the presence of nucleotides did not disrupt the binding of ssDNA and dsDNA with exposed 5′ ends (Table 2), confirming that an exposed 5′ end is required for hSWS1-SWSAP1 binding. Particularly, hSWS1-SWSAP1’s binding to ssDNA and DNA with exposed 5′ ends was maintained regardless of the presence of nucleotides. The binding affinity differences between DNA with exposed 5′ ends and those without dramatically increased in the presence of nucleotides. These results demonstrated that nucleotide binding enhances the DNA-binding specificity of hSWS1-SWSAP1 to DNA exposed 5′ ends. To validate these findings, we repeated the analysis using the Walker A (K18A) and Walker B (D96A) motif mutants of SWSAP1 in the hSWS1-SWSAP1 dimer (Table 2). These key residue mutations, K18A and D96A, specifically abolish the nucleotide-binding and ATP-hydrolysis functions of hSWS1-SWSAP1, respectively. DNA binding of these mutants was verified using EMSAs (Fig. S1*K*). As expected, the nucleotide binding-deficient K18A mutant (Fig. 2*A*) lost nucleotide-induced DNA binding alterations (Table 2 and Fig. S3, *F*–*J*). On the other hand, the D96A mutant, with intact nucleotide binding (Fig. 2*A*), maintained the same nucleotide binding-dependent effects as the wildtype (WT) protein (Table 2 and Fig. S1, *F*–*J*). Therefore, the DNA-binding data confirmed that nucleotide binding increases the specificity of DNA binding. Overall, the DNA-binding analysis indicated that the 5′-end exposure is important for the DNA binding of hSWS1-SWSAP1.

**Table 2:** DNA-binding affinities of hSWS1-SWSAP1 and mutants (K18A, D96A) in the presence of nucleotides

DNA^a^	Type & schematic	Nucleotide	WT *K*_d_ (μM)^b^	K18A *K*_d_ (μM)^b^	D96A *K*_d_ (μM)^b^
ss39		-	11.2 ± 0.4	9.9 ± 0.4	10.4 ± 0.7
		ATP	6.9 ± 0.7	9.5 ± 0.7	7.8 ± 0.4
		ADP	3.2 ± 0.2	7.4 ± 0.2	3.9 ± 0.3
ds39		-	19.2 ± 0.9	12.2 ± 1.0	13.6 ± 0.6
		ATP	-	10.0 ± 0.9	-
		ADP	-	8.73 ± 0.7	-
3′-overhang		-	14.9 ± 0.7	7.1 ± 0.2	11.0 ± 0.8
		ATP	-	7.3 ± 0.6	-
		ADP	-	8.7 ± 0.4	-
Fork		-	7.7 ± 0.5	4.6 ± 0.4	7.4 ± 0.4
		ATP	18.6 ± 0.7	8.2 ± 0.4	22.4 ± 0.4
		ADP	25.6 ± 0.7	7.0 ± 0.8	33.9 ± 0.9
5′-overhang		-	3.6 ± 0.2	3.9 ± 0.3	3.8 ± 0.2
		ATP	3.6 ± 0.1	3.9 ± 0.2	4.3 ± 0.4
		ADP	8.9 ± 0.4	3.4 ± 0.3	5.1 ± 0.2

a Sequence of DNA substrates can be found in Table 1.

b Data were obtained through nonlinear regression of the binding data as described in Experimental procedures and represent the mean ± SD (standard deviation) of three independent experiments. “-” indicates no detectable DNA binding for the substrate.

### hSWS1-SWSAP1 binds ATP/ADP

The ATP-binding and ATP-hydrolysis capabilities of the human Shu complex are vital for its function in promoting HR-associated DNA damage tolerance (7). The Walker A (ATP-binding) and the Walker B (ATP-hydrolysis) motifs are conserved among RAD51 homologs (12, 13, 14, 15) and are both present in SWSAP1 (7). The SWSAP1 subunit has been shown to be responsible for the DNA-stimulated ATPase activity in the hSWS1–SWSAP1 complex (7). We made the Walker A (K18A) and B (D96A) mutations in SWSAP1 and purified the dimer mutants (Fig. 1, *A* and *B*). To characterize the ATP-binding properties of hSWS1-SWSAP1, we performed ATP-binding assays using a fluorescent ATP analog, 2′(3′)-O-(2,4,6-trinitrophenyl) adenosine 5′-triphosphate (TNP-ATP). We measured the changes in TNP-ATP fluorescence intensity after adding hSWS1-SWSAP1 proteins. The WT hSWS1-SWSAP1 increased the fluorescence intensity with a blue shift of the emission peaks from 561 nm (the TNP-ATP fluorescence peak) to 541 nm (Fig. 2*A*). Expectedly, these results indicated that the hSWS1–SWSAP1 complex binds TNP-ATP and sequesters it from the aqueous solution. The Walker A mutant (K18A) abolished the binding of TNP-ATP, while the Walker B mutant (D96A, ATP-hydrolysis-deficient) retained the same nucleotide-binding ability as the WT protein (Fig. 2*A*). Thus, the mutant data confirmed that the nucleotide binding is specifically from hSWS1-SWSAP1.

After the confirmation of nucleotide binding, we determined the binding constant (*K*_d_^TNP-ATP^) of hSWS1-SWSAP1 for TNP-ATP (7.3 ± 0.6 μM) (Fig. 2*B*). Then, we examined the ATP/ADP-binding affinity by using ATP and ADP to compete with TNP-ATP in the binding assays. The addition of ATP or ADP (2.5 mM) in excess of TNP-ATP (5 μM) reduced the fluorescence intensities in hSWS1-SWSAP1, indicating that the nucleotides bind to the specific site competitively (Fig. 2*C*). The fluorescence reductions caused by ATP and ADP competition were essentially equal, indicating no preference for one nucleotide over the other. The binding constant for ATP (*K*_d_^ATP^) was determined to be 72.3 μM (Fig. 2*D*). As intracellular ATP concentration is ∼3 mM (19, 20), the human Shu complex would likely be in the ATP-bound form in cells to readily carry out its HR-associated functions. Additionally, the nucleotide-binding affinity of the human Shu complex is weaker than that of human RAD51 (*K*_d_^ATP^ = ∼5 μM) (21). Thus, RAD51 would likely be saturated with ATP before hSWS1-SWSAP1 in a system containing both RAD51 and the human Shu complex.

### hSWS1-SWSAP1 is a DNA-dependent ATPase that prefers DNA with an exposed 5′ end

Using malachite green-based ATP hydrolysis assays, we first checked the ATPase activity of our purified hSWS1-SWSAP1. The assays revealed that hSWS1-SWSAP1 has a basal level of ATPase activity without DNA (Fig. 2*E*). The activity was elevated in the presence of a DNA substrate with an exposed 5′ end (Fig. 2*E*). As negative controls, both Walker motif mutants completely lost ATPase activity (Fig. 2*E*). Next, we determined the kinetic parameters of the ATPase activity with different DNA substrates (Table 3 and Fig. 2*F*). The catalytic efficiencies (*k*_cat_/*K*_m_) for the DNA substrates possessing an exposed 5′ end (ss39, Fork, and 5′-overhang) were 7.5, 12.5, and 18.0 s^−1^ ⋅ M^−1^, respectively, which were significantly higher than the basal level (1.3 s^−1^ ⋅ M^−1^) (Table 3). Particularly, the dsDNA substrates with an exposed 5′ end and a ssDNA/dsDNA junction enhanced the catalytic efficiency by 10- to 14-fold compared to the basal level of the WT dimer without DNA (Table 3). In contrast, the catalytic efficiencies (*k*_cat_/*K*_m_) of the blunt-ended ds39 (1.9 s^−1^ ⋅ M^−1^) and the 3′-overhang (2.6 s^−1^ ⋅ M^−1^) substrates were close to the basal activity without DNA (1.3 s^−1^ ⋅ M^−1^) (Table 3). The stimulation of ATPase activity by the DNA substrates corresponds to the DNA-binding affinity of the substrates in the presence of ATP (Tables 2 and 3). The results suggest that optimal ATPase activity of hSWS1-SWSAP1 is specifically stimulated by DNA with an exposed 5′ end. Taken together, the importance of such an end structure with respect to the DNA-stimulated ATPase activity of hSWS1-SWSAP1 suggests that this Shu complex homolog has substrate preferences for its ATPase activity. The preference for an exposed 5′ end of dsDNA was also previously reported in the yeast Shu complex (16), which aligns with the strand-specific recognition of lesions on the lagging strand at stalled replication forks (22).

**Table 3:** ATPase activity of hSWS1-SWSAP1

DNA^a^	Type & schematic	*K*_m_ (mM)^b^	*V*_max_ (μM Pi ⋅ min^-1^)^b^	*k*_cat_ (min^-1^)^c^	*k*_cat_/*K*_m_ (s^-1^ ⋅ M^-1^)
-	-	10.0 ± 0.2	0.75 ± 0.01	0.75	1.3
ss39		1.3 ± 0.1	0.57 ± 0.01	0.57	7.5
ds39		5.9 ± 0.2	0.67 ± 0.06	0.67	1.9
Fork		1.3 ± 0.1	0.98 ± 0.02	0.98	12.5
3′-overhang		4.7 ± 0.3	0.73 ± 0.02	0.73	2.6
5′-overhang		0.9 ± 0.1	1.00 ± 0.02	1.00	18.0

a Sequence of DNA substrates can be found in Table 1.

b Data were obtained through nonlinear regression of the ATPase data as described in Experimental procedures and represent the mean ± SD (standard deviation) of three independent experiments.

c *k*_cat_ = *V*_max_/[hSWS1-SWSAP1], where [hSWS1-SWSAP1] = 1 μM.

### hSWS1-SWSAP1 alters accessibility and stability of RAD51-ssDNA filaments

**Figure 3:**
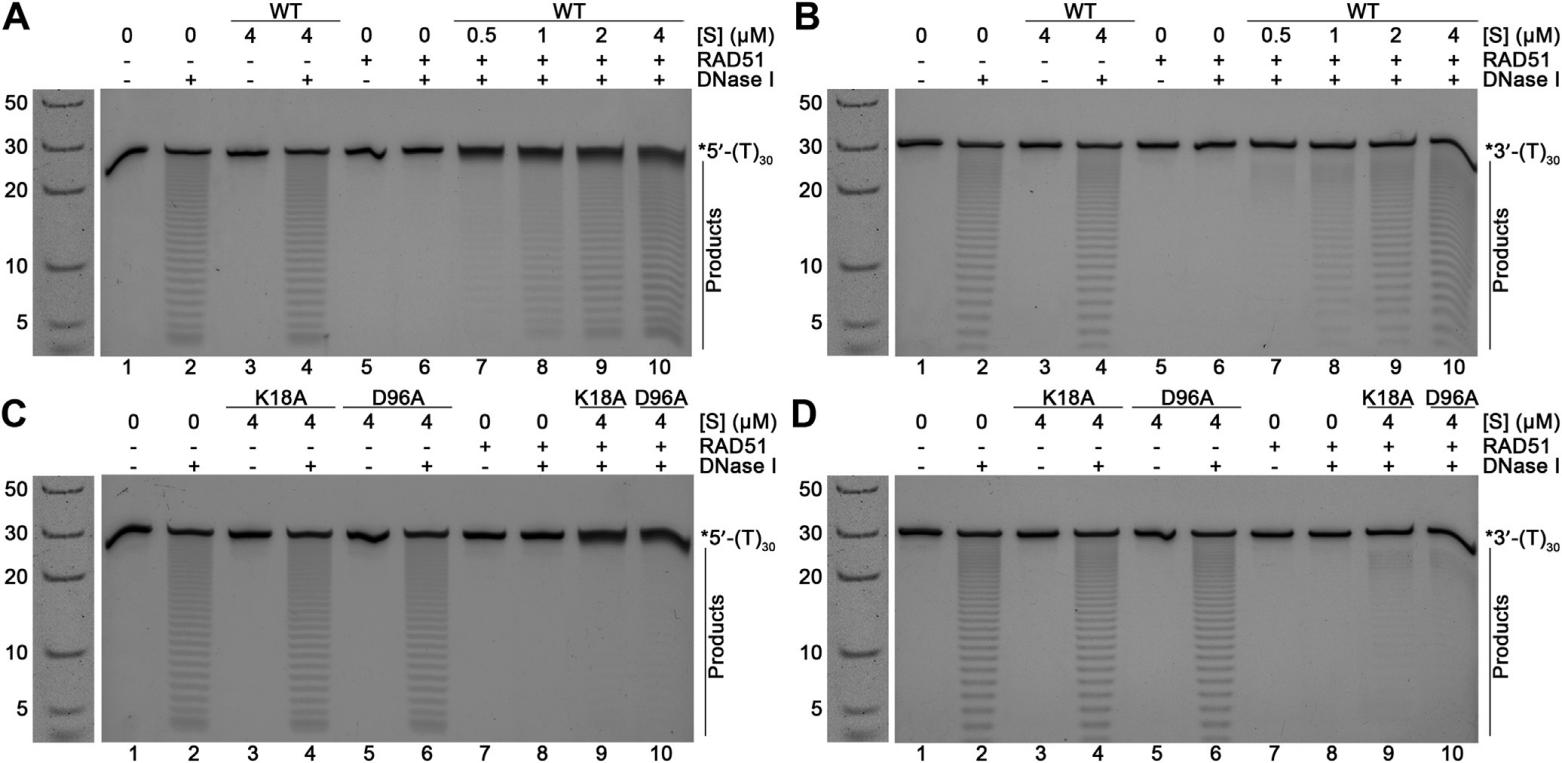
**hSWS1–SWSAP1 complex enhances DNase I digestion on RAD51 filaments**. *A*–*D*, 2-hour DNase I digestion of RAD51-ssDNA filaments labeled on (*A* and *C*) 5′- or (*B* and *D*) 3′-end with 6-FAM and incubated with/without WT hSWS1-SWSAP1 or Walker motif mutants (K18A, D96A). Filaments (2 μM RAD51 on 0.25 μM poly-dT30 ssDNA with 2 mM ATP) were incubated for 10 min and then mixed with increasing concentrations of WT hSWS1-SWSAP1 or mutants ([S]). Digestions were carried out using 2 Unit DNase I at 37 °C. Reaction mixtures were resolved by 22.5% denaturing PAGE. DNA ladder with nucleotide sizes (in nt) is shown to the *left* of each gel and reused across all panels to indicate the sizes of the substrate and its degradation products. “∗5′-(T)_30_” and “∗3′-(T)_30_” stand for 5′- and 3′-labeled poly-dT30 ssDNA, respectively. 6-FAM, 6-carboxyfluorescein; PAGE, polyacrylamide gel electrophoresis; ssDNA, single-stranded DNA.

Recently, the human Shu complex was shown to form complexes with human RAD51 filaments (17). We used DNase I digestion protection assays to probe the changes in RAD51 filaments bound by hSWS1-SWSAP1. Poly-dT ssDNA substrates were used to avoid sequence biases. We incubated purified RAD51 (Fig. S2) with the poly-dT substrates (5′- or 3′-6-FAM-labeled) to form Rad51 filaments. We then conducted two-hour DNase I digestions on RAD51-ssDNA filaments under different conditions. The ssDNA substrates were protected from DNase I digestion by the formation of Rad51-ssDNA filaments, unlike the substrate in the presence of hSWS1-SWSAP1 alone (lane 6 *versus* lanes 2 & 4 in Fig. 3, *A* and *B*). Interestingly, the addition of hSWS1-SWSAP1 enhanced the digestion of both 5′- and 3′-labeled RAD51 filaments in a concentration-dependent manner (lanes 7–10 in Fig. 3, *A* and *B*). The increased sensitivity to DNase I, regardless of fluorophore labeling positions, suggests that hSWS1-SWSAP1 induces conformational changes throughout the entire filament, increasing the accessibility of ssDNA to the nuclease cleavage. To validate these observations, we performed the assays with the Walker motif mutants, which are devoid of either ATP-binding (K18A) or ATPase activity (D96A). The mutants lost the ability to sensitize RAD51 filaments to DNase I digestion (lanes 9 & 10 in Fig. 3, *C* and *D*), indicating the action on Rad51 filament is specifically from hSWS1-SWSAP1 and dependent on its ATP binding and hydrolysis.

**Figure 4:**
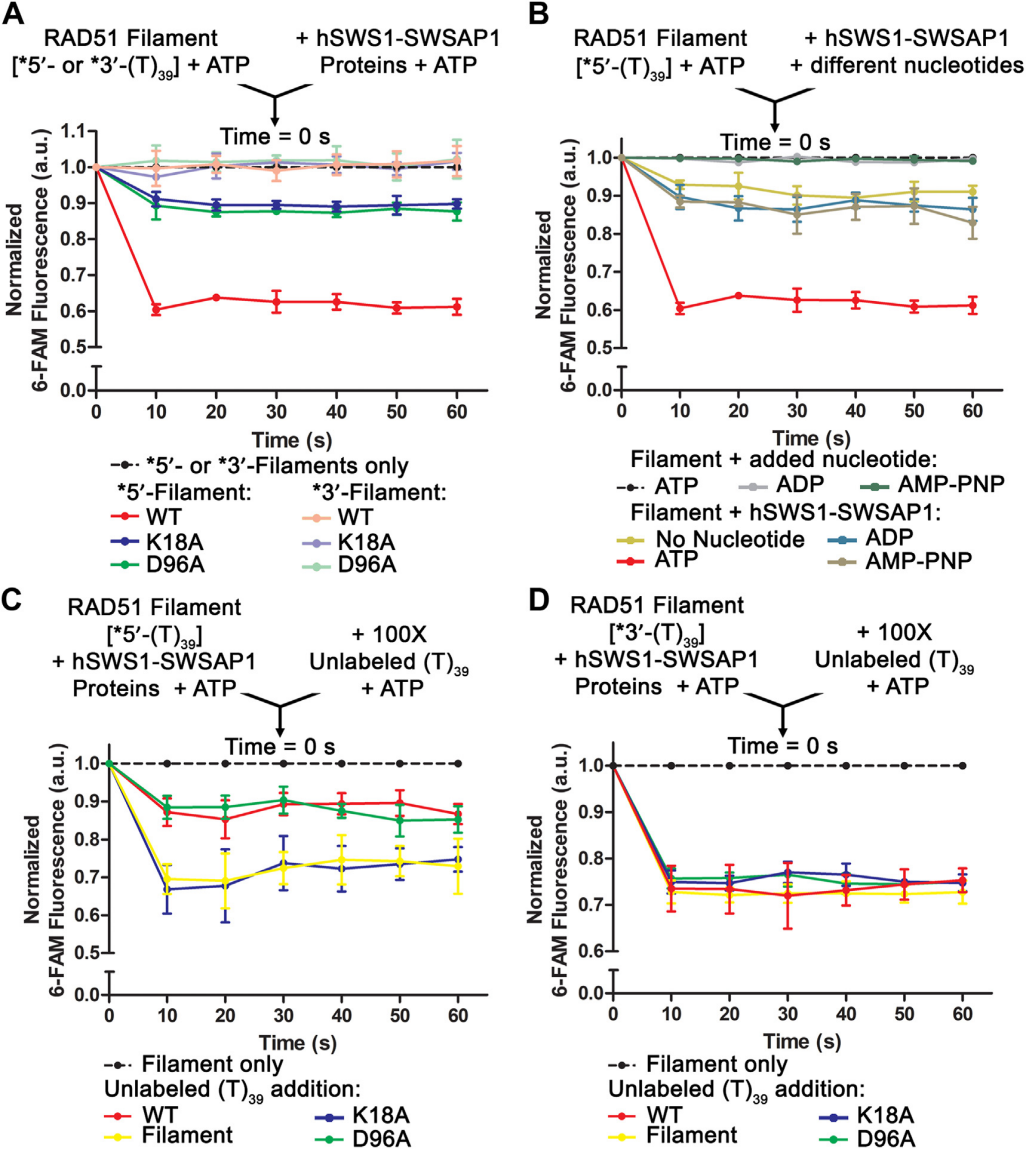
**Conformational and stability changes of RAD51 filaments induced by hSWS1-SWSAP1.***A*, fluorescence intensity profiles of RAD51-ssDNA filaments with/without hSWS1-SWSAP1 proteins over 60-s time frame. Filaments (1 μM RAD51 on 15 nM 5′- or 3′-labeled poly dT39 ssDNA) were incubated for 10 min and then mixed with WT hSWS1-SWSAP1 or Walker motif mutants (50 nM each), all in the presence of ATP (2 mM). *B*, fluorescence intensity profiles of RAD51-ssDNA filaments (preincubated with 2 mM ATP) mixed with hSWS1-SWSAP1 (preincubated with 2 mM of the indicated nucleotide), over 60 s. *C* and *D*, fluorescence intensity profiles of RAD51-ssDNA filaments with/without WT hSWS1-SWSAP1 and Walker motif mutants, mixed with 100-fold excess of unlabeled ssDNA, over 60 s. Samples were excited at 490 nm, and emission readings were measured at 522 nm at equilibrium (37 °C). Filament-alone data were normalized as the baseline in arbitrary units corresponding to fluorescence intensities (*dashed black lines*). “∗5′-(T)_39_” and “∗3′-(T)_39_” stand for 5′- and 3′-labeled poly-dT39 ssDNA, respectively. 6-FAM, 6-carboxyfluorescein; ssDNA, single-stranded DNA.

We measured fluorescence intensities of the RAD51 filaments after the additions of hSWS1-SWSAP1 proteins at 10-s intervals over a 60-s time frame to examine the initial effects of hSWS1-SWSAP1 on the filaments. Without hSWS1-SWSAP1, the RAD51 filaments had stable fluorescence intensities, which were normalized as a baseline (dashed black line in Fig. 4*A*). The fluorescence intensities of the 5′-6-FAM-labeled RAD51-ssDNA filaments were reduced by ∼40% within 10 s after hSWS1-SWSAP1 addition (red line in Fig. 4*A*), while no reduction was observed in the 3′-labeled sample within the 60-s time frame (pink line in Fig. 4*A*). The fluorescence reduction resulted from increased solvent exposure of the fluorophore and suggests conformational changes of the filaments. The fluorescence changes of the filaments occurred only at the 5′ end and not at the 3′ end within the 60-s time frame (Fig. 4*A*). Thus, the data suggest that the action of hSWS1-SWSAP1 is initiated at the 5′ end of the filaments. This polarity of action on the filaments is consistent with those observed in the *C*. *elegans* and yeast Shu homologs (16, 18). Furthermore, the Walker A (ATP-binding-deficient) and Walker B (ATP-hydrolysis-deficient) mutants, as controls, lost the majority of the fluorescence changes of 5′-labeled RAD51 filaments (Fig. 4*A*). The fluorescence data indicate that conformational changes of the filaments by hSWS1-SWSAP1 initially occur at the 5′ end. The dependence on ATP binding and hydrolysis of hSWS1-SWSAP1 is consistent with the digestion data above.

To further validate the requirement of ATP hydrolysis, we measured the fluorescence change of 5′-labeled RAD51 filaments upon mixing with WT hSWS1-SWSAP1 that was preincubated with ATP, ADP (hydrolysis product), or AMP-PNP (nonhydrolyzable), as described (18). The hSWS1-SWSAP1 preincubated with ADP/AMP-PNP and its nucleotide-free form lost the majority of fluorescence reduction (Fig. 4*B*), which is comparable to those of the Walker motif mutants (Fig. 4*A*). This indicates that the action of hSWS1-SWSAP1 on the conformation of RAD51 filaments is specifically dependent on ATP hydrolysis.

We then explored the roles that ATP binding and hydrolysis of hSWS1-SWSAP1 play in filament stability. We measured the fluorescence changes of the RAD51 filaments when mixed with 100-fold of unlabeled ssDNA counterpart over 60 s. Addition of excess unlabeled ssDNA reduced the fluorescence intensities of RAD51 filaments by ∼30% in 10 s and remained constant (yellow lines in Fig. 4, *C* and *D*). Fluorescence reduction represents exchanges of labeled ssDNA in the RAD51-ssDNA filament with the unlabeled ssDNA. In the presence of hSWS1-SWSAP1, the fluorescence reduction by the excess of unlabeled DNA was inhibited for the 5′-labeled substrate but remained unchanged for the 3′-labeled substrate (red lines in Fig. 4, *C* and *D*). The results indicate that hSWS1-SWSAP1 reduces DNA exchanges at the 5′ end but does not significantly affect the 3′ end in the 60-s time frame. The observations highlight the initial 5′-end preference of hSWS1-SWSAP1 to stabilize RAD51 filaments, which was also shown in the *C*. *elegans* and yeast Shu homologs (16, 18). The fluorescence assays were also carried out with the Walker motif mutants, both with impaired ATPase activity. The ATP-binding mutant (K18A) lost the influence on RAD51 filament stabilization (blue lines in Fig. 4*C*). In contrast, the ATP-hydrolysis mutant (D96A), with intact ATP binding, stabilized filaments at the 5′ end similar to the WT protein (*green* and *red* lines in Fig. 4*C*). These results suggest that filament stabilization is dependent on ATP binding, but not ATP hydrolysis, by hSWS1-SWSAP1. Taken together, the human Shu complex stabilizes RAD51 filaments in an ATP binding–dependent manner and alters the conformation of the filaments with its ATPase activity.

## Discussion

Biochemical characterization of the human Shu complex provides valuable insights into its molecular role of modulating RAD51 filament properties in HR. Our DNA-binding analysis of hSWS1-SWSAP1 showed that it binds DNA weakly (*K*_d_: 3.6–43.7 μM) compared to RAD51, which binds DNA with a *K*_d_ of ∼0.1 μM (23, 24). The low DNA-binding affinity of hSWS1-SWSAP1 fits its role as a mediator of RAD51 filaments, instead of a DNA-binding competitor which displaces RAD51 from DNA (17). Additionally, RAD51 is likely more abundant than hSWS1-SWSAP1 in cells, which would further emphasize its predominant DNA binding over hSWS1-SWSAP1 *in vivo*.

**Figure 5:**
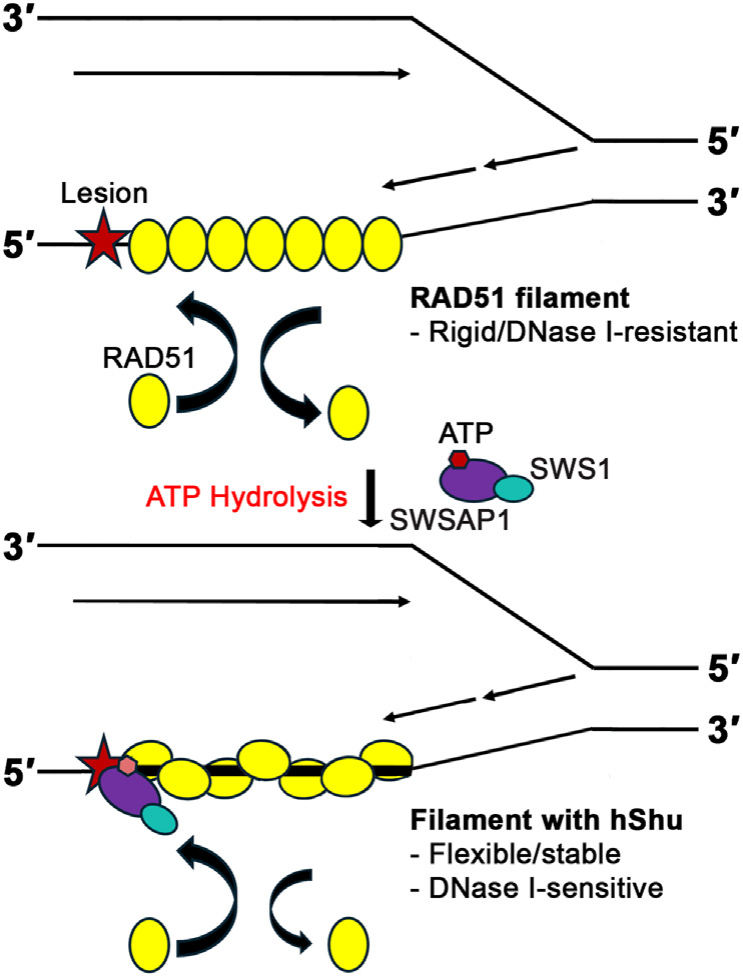
**Molecular model for the action of hSWS1-SWSAP1 on RAD51-ssDNA filaments.** The role of hSWS1-SWSAP1 (hShu) in homologous recombination as a DNA-stimulated ATPase. The hShu complex binds to the 5′ end of a RAD51 filament containing DNA lesions, such as abasic lesions, stabilizing the filament in an ATP-dependent manner. The alteration of the RAD51 filament conformation is dependent on the ATPase activity of hSWS1-SWSAP1, rendering the filament flexible and “open” for HR-associated processes. HR, homologous recombination.

More importantly, hSWS1-SWSAP1 binds DNA with an exposed 5′ end preferentially, particularly in the presence of adenine nucleotides. Under replication stress induced by DNA damage, the yeast Shu complex facilitates HR-associated error-free lesion bypass by acting on the lagging strand at stalled replication forks (22). The 5′-end preference observed here indicates that the human Shu complex also specifically recognizes the lagging strand structure, which presents an exposed 5′ end for lesion bypass (Fig. 5). Therefore, this selective binding to the 5′ ends of ssDNA and RAD51 filaments fits its proposed role in promoting HR at DNA lesions. Notably, our experiments were conducted using undamaged DNA substrates. The observed binding preference is likely an inherent property of hSWS1-SWSAP1, which would facilitate the recognition of lesioned DNA on the lagging strand. The DNA-binding preference for exposed 5′ ends of hSWS1-SWSAP1 is also observed in the yeast and *C*. *elegans* homologs (18, 22), implying an important evolutionarily conserved feature. Furthermore, the 5′-end preference from our DNA-binding data is consistent with the DNA-stimulated ATPase activity data. In addition, RAD51 is also a DNA-stimulated ATPase (25) that hydrolyzes ATP during initial filament assembly (26). Therefore, using ATPase activity to regulate their function is common to RAD51 and its paralogs.

Our work quantitatively characterized the ATP-binding and ATP-hydrolysis properties of the human Shu complex. The K18A mutation abolished the ATP binding of hSWS1-SWSAP1 and, in turn, eliminated the ATPase activity, while the D96A mutant abolished the ATPase activity and retained intact ATP binding. The WT and mutant proteins allowed us to study hSWS1-SWSAP1 for its action on RAD51 filaments and explore the regulatory roles of ATP binding and hydrolysis in RAD51 filament modulation. hSWS1-SWSAP1 has been shown to facilitate RPA dissociation from ssDNA prior to RAD51 filament formation (17). Our findings revealed a subsequent role in modulating the conformation of pre-assembled RAD51 filaments. Specifically, we demonstrated that hSWS1-SWSAP1 induces ATP hydrolysis--dependent conformational changes in RAD51 filaments, initiating at the 5′ end and propagating along the filament toward the 3′ end (Fig. 5). Recently, the hSWS1–SWSAP1 complex was shown to form complexes with RAD51 filaments, resulting in interspersed filaments, under conditions where ATP hydrolysis is inhibited by Ca^2+^ (17). We specifically inhibited hSWS1-SWSAP1’s ATPase activity with Walker box mutations to reveal that hSWS1-SWSAP1 can stabilize filaments in an ATP binding–dependent manner. We demonstrated that the filament stabilization by hSWS1-SWSAP1 is specifically dependent on ATP binding (Fig. 5). Overall, the hSWS1-SWSAP1–enhanced accessibility and stability of RAD51 filaments likely contribute to its ability to facilitate subsequent DNA damage bypass *via* HR (Fig. 5). In closing, our work reveals that the human Shu complex utilizes its DNA-binding specificity, nucleotide-binding affinity, and ATPase activity in concert to regulate RAD51 filament properties in HR for the DNA damage response.

## **Experimental procedures**

### Generation of recombinant bacmids for insect cell protein expression

The cDNA encoding human *SWS1* and human *SWSAP1* was kindly provided by Dr. Jun Huang of the Life Sciences Institute, Zhejiang University. We built all the plasmid constructs using the polymerase incomplete primer extension method (27). The protocol for generating recombinant bacmids was adapted from the MultiBac Kit (Geneva Biotech) manual, with modifications. Briefly, *SWS1* and *SWSAP1* were cloned into the modified pACEBac1 vector for expression of SWSAP1 with a TEV-cleavable 6xHis tag and native hSWS1. The K18A (Walker A) and D96A (Walker B) mutants of SWSAP1 were generated for function validation. Transfer vectors of pACEBac1-6xHis-TEV-SWSAP1-hSWS1 and its Walker motif mutants were transformed into *Escherichia coli* competent cells harboring the DH10EMBacY bacmid. Tn7 transposition integrated genes of interest into the recipient baculovirus. Recombinant bacmids carrying the integrated multigene cassettes were selected by blue/white screening and gentamycin resistance. Isolated recombinant bacmids with genes of interest were verified using PCR analysis, as outlined in the Bac-to-Bac Baculovirus Expression System (Invitrogen) manual.

### Expression and purification of hSWS1-SWSASP1 and RAD51

The hSWS1-SWSAP1 dimer and its mutants were expressed in insect cells as described (28), with modifications. Prior to transfection, Sf9 cells were maintained in Gibco Sf900-II SFM (Thermo Fisher Scientific) at a density of 1.0 to 4.0 × 10^6^ cells/ml with viability of 95% or higher. EMBacY-6xHis-TEV-SWSAP1-hSWS1 recombinant bacmids were heat-sterilized at 55 °C for 1 h. The bacmid-polyethyleneimine mixture, incubated at room temperature for 30 min, was added dropwise to the diluted Sf9 cell culture. Transfected Sf9 cell culture was incubated at 27 °C with shaking. Five days postinfection, the medium containing recombinant baculovirus was collected and mixed with fetal bovine serum.

High Five cells were maintained in I-MAX insect cell culture media (Wisent Bio Products) at a density of 1.0 to 4.0 × 10^6^ cells/ml with viability of 95% or higher and infected with the recombinant baculovirus. Cell cultures were harvested by centrifugation 3 days postinfection or at 85% cell viability. Cell paste was resuspended and washed with 1× phosphate-buffered saline buffer.

Cell paste was lysed in a lysis buffer (50 mM Tris-HCl, pH 7.5, 0.5 M NaCl, 5% glycerol, 1 mM PMSF, 1 mM benzamidine, 1 mM E-64, 1 mM bestatin, 1 mM pepstatin A, and 5 mM βMe). Clarified cell lysate was purified using a Ni-HiTrap FF column (GE Healthcare). Then, tag-cleaved proteins were purified by gel filtration chromatography using a Superdex 200 10/300 Gl column (GE Healthcare) and stored in a storage buffer (30 mM Tris-HCl, pH 8.0, 0.25 M NaCl, and 2 mM DTT). The purified proteins were of high purity (>95%) and homogeneity as determined by Bis-Tris-SDS-PAGE.

The plasmid pCH1/RAD51o (plasmid #102562), encoding human *RAD51*, was purchased from Addgene. The plasmid was transformed into *E*. *coli* Acella cells (Edge Bio) with a RecA deletion for overexpression. Cell cultures were grown to an *A*_600_ of 0.6 to 0.7, induced at 37 °C for 4 h, harvested by centrifugation, and stored at −80 °C until protein purification at 4 °C.

Cell paste was lysed in a lysis buffer (50 mM Tris-HCl, pH 7.5, 0.5 M NaCl, 5% glycerol, 1 mM PMSF, 1 mM benzamidine, 1 mM E-64, 1 mM bestatin, 1 mM pepstatin A, and 5 mM βMe). RAD51 protein was isolated from the clarified cell lysate by ammonium sulfate precipitation. The resulting pellet was resuspended in the lysis buffer and dialyzed against a dialysis buffer (0.1 M NaCl, 25 mM Tris, pH 7.5, 5% glycerol, and 5 mM βMe). Dialyzed sample was purified with HiTrap Q FF (GE Healthcare) and Hitrap Heparin HP (GE Healthcare) columns. RAD51 protein was eluted by a linear gradient of NaCl. The purified RAD51 was stored in storage buffer (0.15 M NaCl, 20 mM Hepes, pH 7.5, 10% glycerol, 1 mM EDTA, and 1 mM DTT).

Protein concentrations were quantified using the Bio-Rad Protein Assay Dye Reagent in a colorimetric assay. Final protein concentrations were determined from a standard curve generated using bovine serum albumin (New England Biolabs).

### EMSAs for DNA binding

DNA-binding reactions were carried out with increasing concentrations of hSWS1-SWSAP1 proteins and 0.05 μM 6-FAM-labeled DNA substrate. The protein–DNA mixtures were resolved in native polyacrylamide gels and captured by the ChemiDoc MP Imaging System (Bio-Rad). Assays were performed in triplicate.

### FPAs for DNA binding

FPAs for DNA-binding analysis of hSWS1-SWSAP1 were conducted as described previously (16, 29, 30). Fluorescence polarization measurements were recorded using a VICTOR^3^ V 1420 Multilabel Counter (PerkinElmer) with an integrated polarizer. Reactions were carried out at 37 °C. Each reaction mixture contained 5 nM 6-FAM-labeled DNA substrates with increasing concentrations of hSWS1-SWSAP1 proteins. Changes in fluorescence polarization were obtained from subtracting each measurement by that of DNA alone. Triplicate data were fitted using a one-site binding model, in PRISM5 (GraphPad), to a quadratic (Equation 1) to determine the *K*_d_ values. DNA concentrations in molecules were converted to concentrations in nucleotides for curve fitting.

(1) $$Y=\frac{M\left(\left(x+D+K_{d}\right)-\sqrt{{\left(x+D+K_{d}\right)}^{2}-\left(4Dx\right)}\right)}{2D}$$

### Analysis of protein–ATP interactions with a fluorescent ATP analog

TNP-ATP spectra, for ATP-binding analysis of hSWS1-SWSAP1, were collected under conditions described previously (16, 31). Experiments were performed using a Synergy H1 Hybrid Multi-Mode Microplate Reader (BioTek Instruments) at 37 °C. For the TNP-ATP saturation assays, emission readings at 540 nm were collected for 2 μM hSWS1-SWSAP1 with increasing TNP-ATP concentrations. The correction factors for the inner filter effect were established and applied to data points above 10 μM TNP-ATP as previously described (32, 33, 34). Triplicate data were fitted in PRISM5 (GraphPad) by nonlinear regression with a one-site*–*specific binding model to determine *K*_*d*_^*TNP-ATP*^, using the (Equation 2).

(2) $$Y=\frac{B_{\max}X}{K_{d}+X}$$

For the competitive binding curve, 2 μM hSWS1-SWSAP1 with fixed 2.5 μM TNP-ATP was titrated with increasing concentrations of ATP. Triplicate data were fitted in PRISM5 (GraphPad) by nonlinear regression with a one-site competitive binding model to determine the *K*_i_^ATP^, using the (Equations 3 and 4).

(3) $$\log\;EC50=\log\;10^{K_{i}}\left(1+\frac{\text{HotNM}}{\text{Hot}K_{d}\text{NM}}\right)$$

(4) $$Y=\text{Bottom}+\frac{\text{Top}-\text{Bottom}}{1+10^{X-\log\;EC50}}$$

The competitive binding dataset was then used to derive the corresponding *K*_*d*_^*ATP*^ parameter, using the (Equations 5 and 6) (35).

(5) $$\alpha =1+\frac{\left[\text{TNP}\;\text{ATP}\right]}{K_{d}^{\text{TNP}\;\text{ATP}}}$$

(6) $$1-\frac{F-F_{\min}}{F_{\max}-F_{\min}}=\frac{\left[ATP\right]}{\alpha K_{d}+\left[ATP\right]}$$

### ATPase activity assays

ATPase activity assays of hSWS1-SWSAP1 were performed as described (16, 36, 37), with slight modifications. The assays were performed with 1 μM hSWS1-SWSAP1 proteins in the presence and absence of 10 μM DNA at 37 °C.

Steady-state ATPase kinetic assays with hSWS1-SWSAP1 were performed with/without DNA (1 μM), 1 μM protein, and increasing concentrations of ATP. Triplicate datasets were used to calculate the kinetic parameters *K*_m_, *V*_max_, and *k*_cat_ with a nonlinear regression fit of the nanomoles Pi released/min to the Michaelis–Menten (Equation 7).

(7) $$Y=\frac{V_{\max}X}{K_{m}+X}$$

### Nuclease protection assays

DNase I digestion assays were performed as described (16). The 5′- or 3′-6-FAM-labeled ssDNA substrates (0.25 μM) were mixed with hSWS1-SWSAP1 proteins. After 30 min incubation at 24 °C, 1 mM CaCl_2_ and 2 Unit bovine pancreatic DNase I (New England Biolabs) were added to protein–DNA mixtures at 37 °C. For reactions with human RAD51 filaments, RAD51-ssDNA was incubated for 10 min at 24 °C prior to the addition of hSWS1-SWSAP1 proteins and further incubated for 30 min. Reaction mixtures were stopped by the addition of a mixture containing EDTA and formamide and subsequently resolved in denaturing polyacrylamide urea gels. Gel images were captured by the ChemiDoc MP Imaging System (Bio-Rad). Assays were performed in triplicate.

### Fluorescence assays of hSWS1-SWSAP1-Rad51 filaments

Fluorescence assays of RAD51 filaments with hSWS1-SWSAP1 were performed as described (16). Samples were excited at 490 nm, and fluorescence measurements were collected at the emission wavelength of 522 nm at 37 °C, using a Synergy H1 Hybrid Multi-Mode Microplate Reader (BioTek Instruments). Reactions consisted of human RAD51-ssDNA filaments (1 μM RAD51 and 15 nM of 5′- or 3′-6-FAM-labeled poly-dT39 ssDNA), 2 mM ATP, and the hSWS1-SWSAP1 proteins (50 nM). For reactions involving nucleotide preincubation of hSWS1-SWSAP1, the ATP, ADP, or AMP-PNP were incubated with hSWS1-SWSAP1. Nucleotide-preincubated hSWS1-SWSAP1 or each individual nucleotide type (negative controls) was mixed with ATP-bound RAD51 filaments after the initial 10-min incubation. In competition assays, a 100-fold excess of unlabeled poly-dT39 ssDNA (1.5 μM) was mixed with the RAD51 filament/hSWS1-SWSAP1 mixture after the initial 10-min incubation. Fluorescence readings of labeled ssDNA were used for background subtraction of all datasets. The initial fluorescence measurement for the preformed RAD51-ssDNA filament alone was used as the 0-s time point. Subsequent time-point measurements were used to normalize all datasets.

## Data availability

Source data are available in supporting information or upon request.

## Supporting information

This article contains supporting information.

## Conflict of interest

The authors declare that they have no conflicts of interest with the contents of this article.
