# Supplementary material for: The role of human Shu complex in ATP-dependent regulation of RAD51 filaments during homologous recombination–associated DNA damage response
Source: J Biol Chem. 2025 May 8;301(6):110212. doi: 10.1016/j.jbc.2025.110212 (PMC12167799; doi:10.1016/j.jbc.2025.110212)
Supplement: Supporting information [file mmc1.docx]

**Supporting Information for**

**The role of human Shu complex in ATP-dependent regulation of RAD51 filaments during homologous recombination-associated DNA damage response.**

Sam S. H. Chu, Guangxin Xing, Hong Ling

Hong Ling

Email: [hling4@uwo.ca](mailto:hling4@uwo.ca)

**This PDF file includes:**

Figures S1 to S3


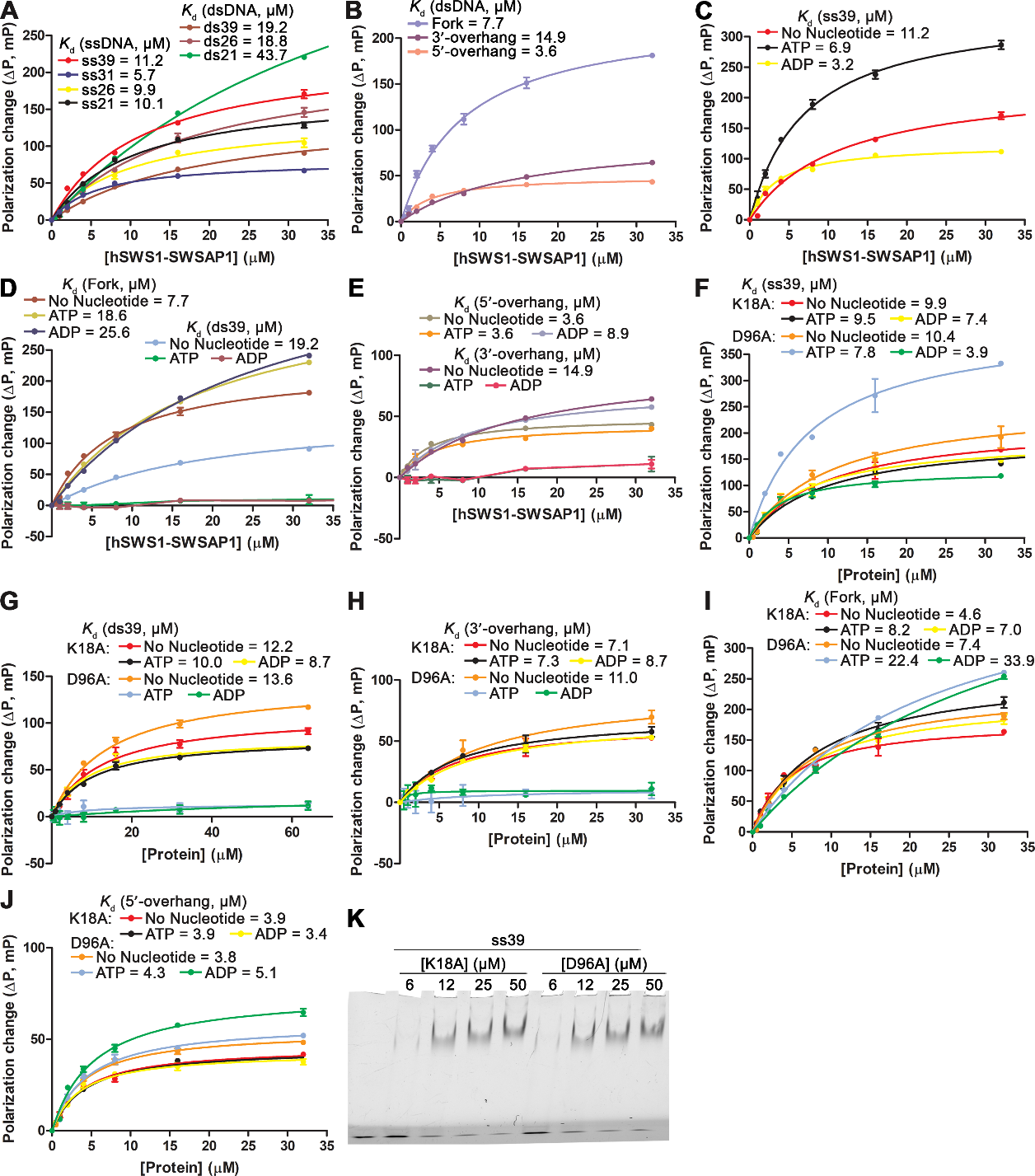


**Figure S1. DNA-binding of hSWS1-SWSAP1 proteins.** (**A, B**) Fluorescence polarization assay (FPA) for DNA binding of WT hSWS1-SWSAP1 with different ssDNA and dsDNA substrates (5 nM). (**C–J**) FPA for DNA binding of (**C–E**) WT hSWS1-SWSAP1 and (**F–J**) Walker motif mutants (K18A, D96A) with different DNA substrates (5 nM), in the presence of adenine nucleotides (2 mM each). Dissociation constants (*K*_d_) in **A–J** were determined by non-linear curve fitting to a one-site binding model (details in **Experimental procedures**). Data represent the mean of three independent replicates, with error bars indicating the standard deviation from triplicate experiments. (**K**) Electrophoretic mobility shift assay (EMSA) for mutant hSWS1-SWSAP1-DNA interactions. Increasing concentrations of the Walker motif mutants of hSWS1-SWSAP1 (K18A, D96A) were mixed with 0.05 μM ss39 DNA substrate. Protein-DNA mixtures were resolved by 2-layer PAGE gels: 5% native polyacrylamide at the top and 15% at the bottom (dark layer). Assays were performed in triplicate with comparable results (**Figure S3D**).

**
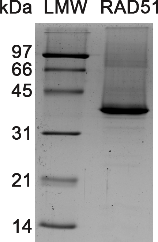
**

**Figure S2. Purified human RAD51 protein.** SDS-PAGE analysis of the purified human RAD51. “LMW” stands for low molecular weight protein ladder.


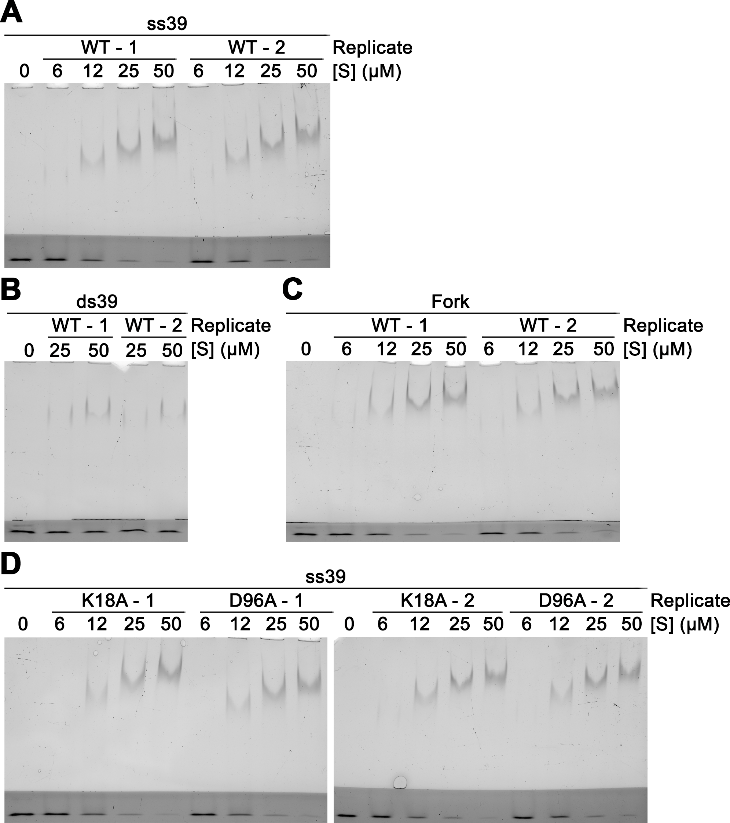


**Figure S3. Electrophoretic mobility shift assay (EMSA) replicates for hSWS1-SWSAP1-DNA interactions.** (**A–D**) Increasing concentrations of (**A–C**) WT hSWS1-SWSAP1 and (**D**) Walker motif mutants (K18A, D96A) ([S]) were mixed with 0.05 μM DNA substrates (ss39, ds39, Fork). Protein-DNA mixtures were resolved by 2-layer PAGE gels: 5% native polyacrylamide at the top and 15% at the bottom (dark layer).
